# Spontaneous disruption of the dividing membrane in monochorionic diamniotic twin pregnancy

**DOI:** 10.1002/ccr3.3437

**Published:** 2020-10-25

**Authors:** Nobuko Yokoyama, Kei Sagawa, Miwa Miyazaki, Shunji Suzuki

**Affiliations:** ^1^ Deaprtment of Obstetrics and Gynecology Japanese Red Cross Katsushika Maternity Hospital Tokyo Japan

**Keywords:** a film‐like image, monochorionic diamniotic twin pregnancy, spontaneous disruption of dividing membrane, ultrasonography

## Abstract

Spontaneous disruption of the dividing membrane in MD twins is rare and be associated with a higher incidence of prematurity and neonatal morbidity. In the present case, a spontaneous disrupted dividing membrane could be noted by ultrasonography.

## Graphical abstract

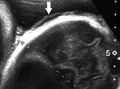

A monochorionic diamniotic (MD) twin pregnancy had progressed uneventfully until 32 weeks of gestation based on weekly ultrasonic examinations, and the dividing membrane could not be demonstrated between the twins by ultrasonography (Figure 1). At 33 weeks of gestation, however, the dividing membrane could not be demonstrated, and a fluffy, film‐like image was detected near the head of the second twin (Figure 2). Emergency cesarean section was performed on the next day because of the presence of repeated variable decelerations on cardiotocogram (Figure 3). Two healthy female infants with loosely entangled umbilical cords were delivered from a single gestational sac. Macroscopic examination showed a falciform remnant of the disrupted in the placenta (Figure 4).

**FIGURE 1:**
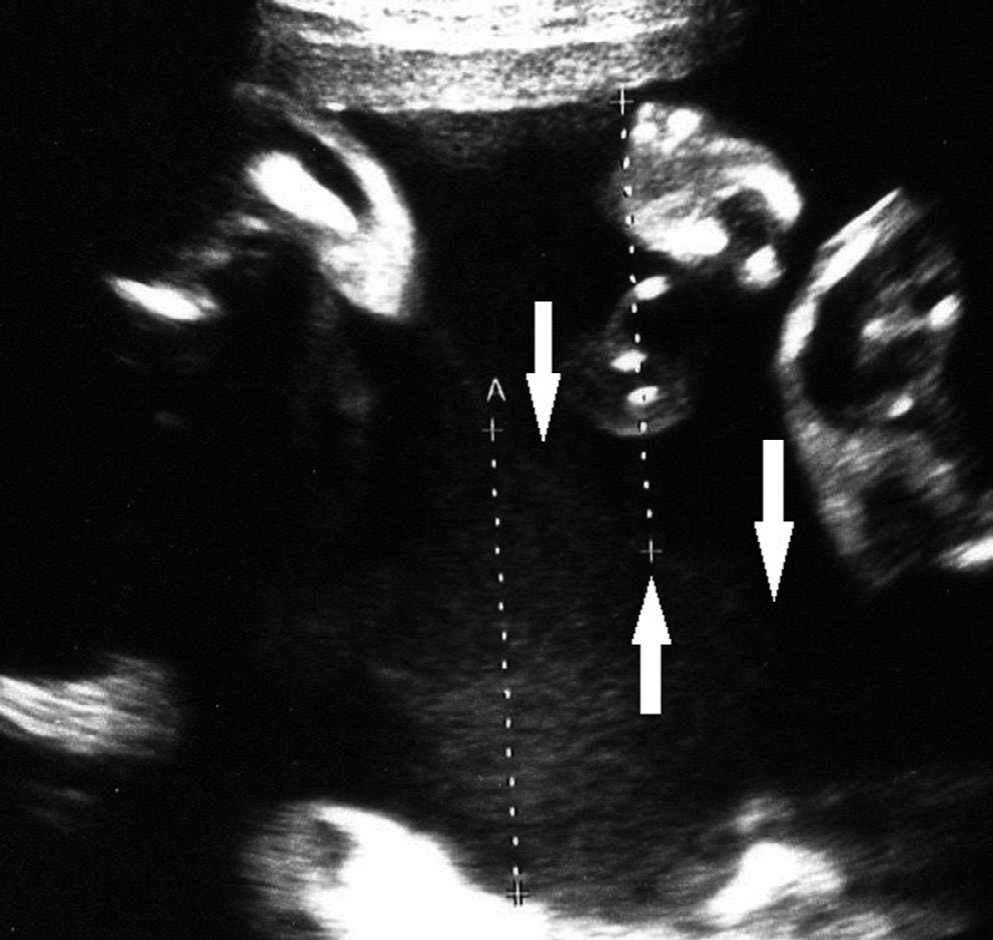
The presence of dividing membrane demonstrated between the twins by ultrasonography at 32 wkeeks of gestations (white arrows)

**FIGURE 2:**
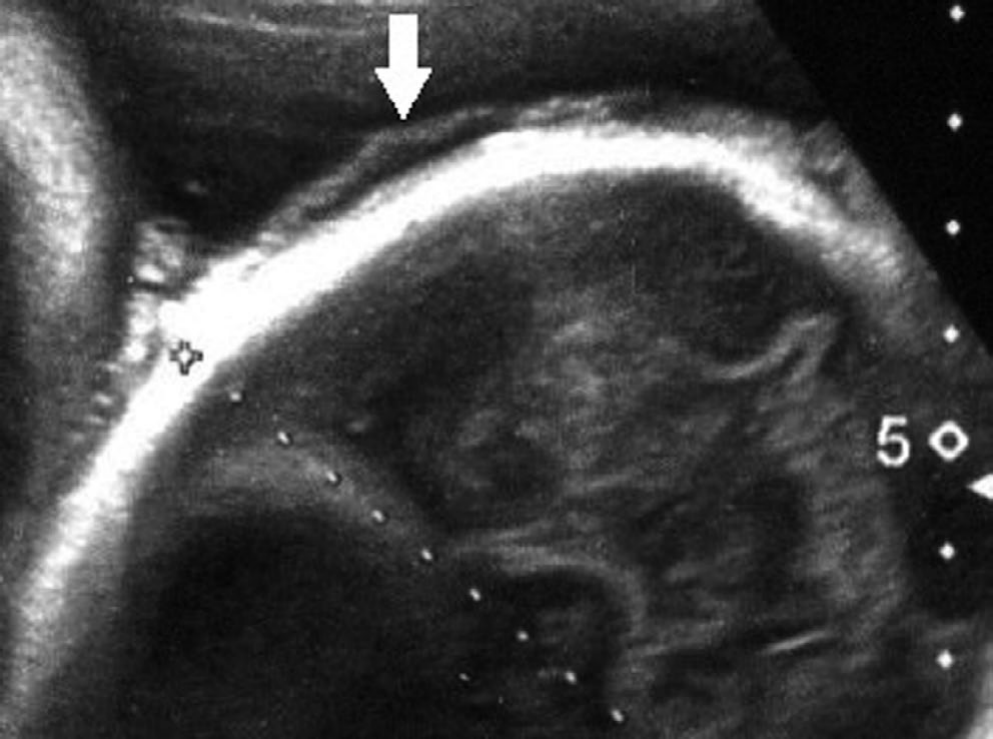
A fluffy, film‐like image detected near the head of the second twin by ultrasonography at 33 wk of gestation (white arrow)

**FIGURE 3:**
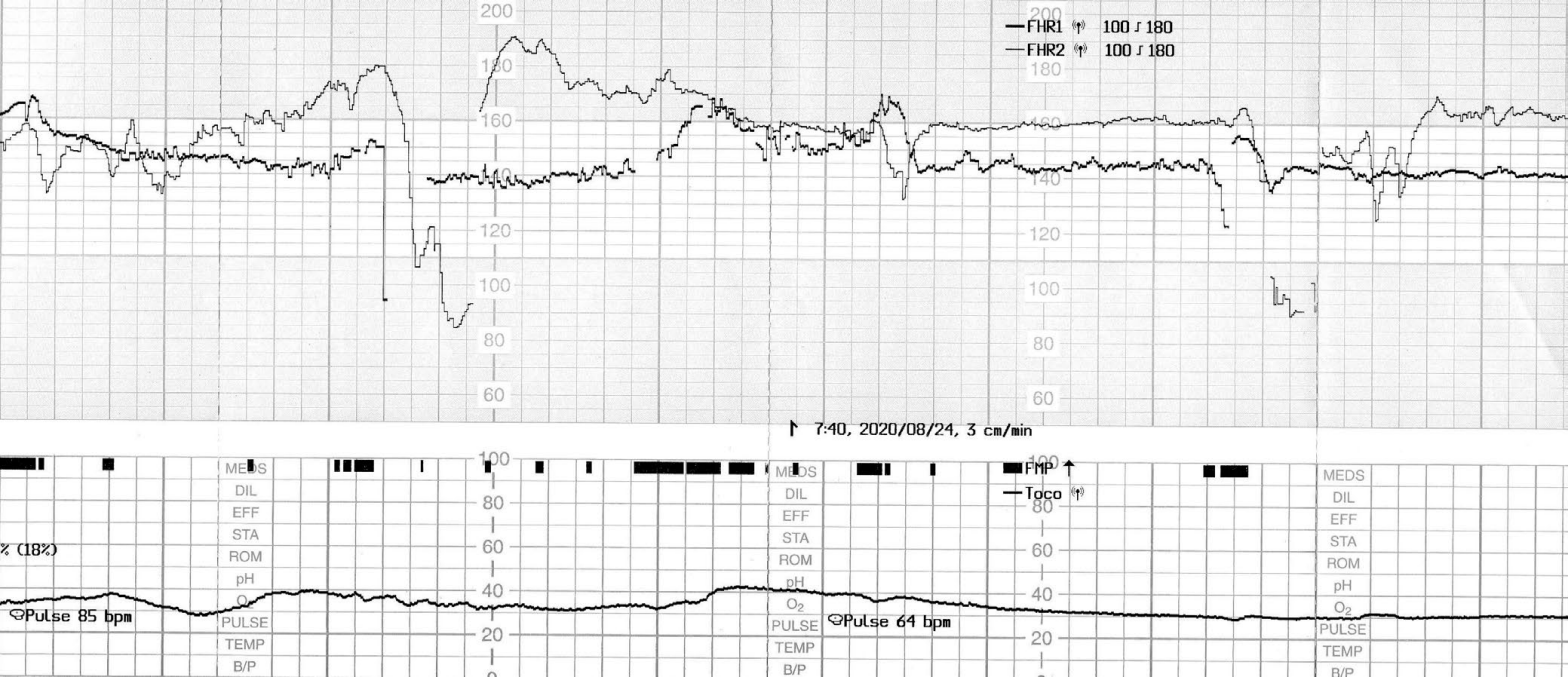
The presence of repeated variable decelerations on cardiotocogram at 33 wk of gestation

**FIGURE 4:**
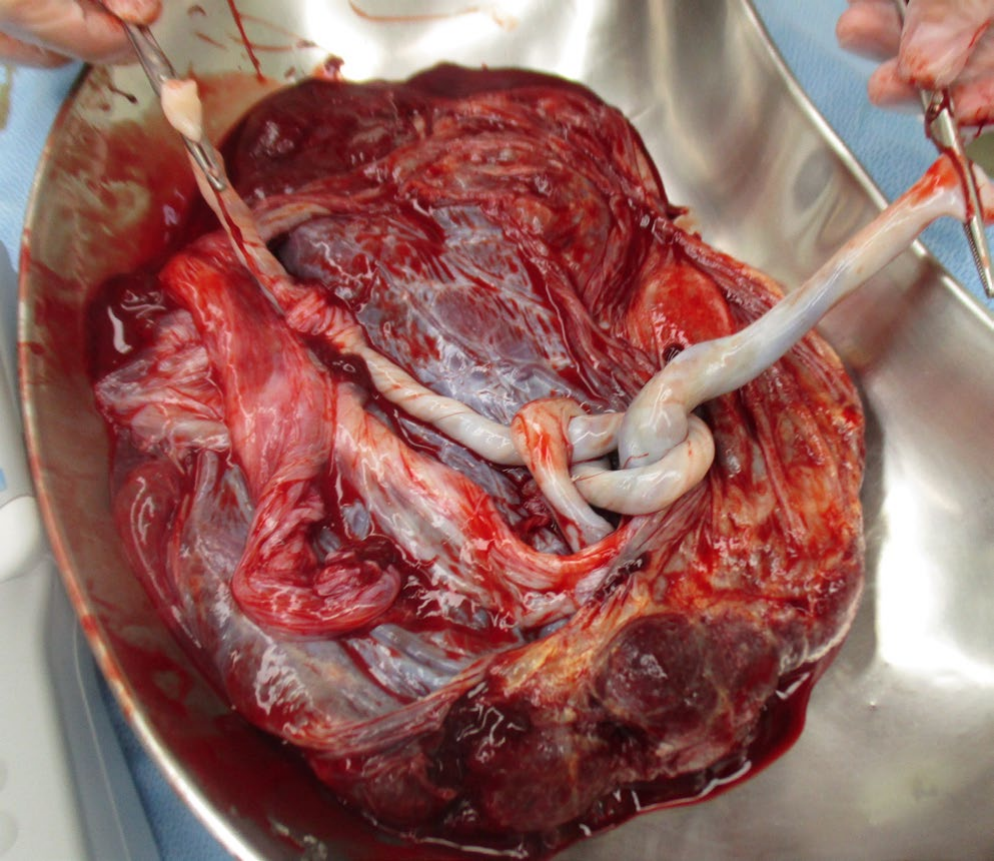
Cord entanglement and falciform remnant of the disrupted dividing membrane in the placenta

Spontaneous disruption of the dividing membrane in MD twin pregnancy may be very rare unlike the cases following laser surgery, but it may increase neonatal morbidity.^1^, ^2^ It may be very important to actively search for signs of the disruption in MD twin pregnancy even if laser surgery is not performed.

## CONFLICT OF INTEREST

No conflict of interest to declare.

## AUTHORS' CONTRIBUTIONS

NY (Primary author and outpatient physician): analyzed the data from the ultrasonographic examination, and wrote and revised the manuscript. KS and MM (Primary care physicians in hospital): performed cesarean section and examined the placenta. SS (Director of the Department): was involved in the conception of the study, analyzed the data comprehensively, and drafted and revised the manuscript.

## ETHICAL APPROVAL

Approval by the Ethical Committee of the Japanese Red Cross Katsushika Maternity Hospital was obtained.
